# A human genome-wide library of local phylogeny predictions for whole-genome inference problems

**DOI:** 10.1186/1471-2164-9-389

**Published:** 2008-08-18

**Authors:** Srinath Sridhar, Russell Schwartz

**Affiliations:** 1 Computer Science Department, Carnegie Mellon University, USA; 2 Department of Biological Sciences, Carnegie Mellon University, USA

## Abstract

**Background:**

Many common inference problems in computational genetics depend on inferring aspects of the evolutionary history of a data set given a set of observed modern sequences. Detailed predictions of the full phylogenies are therefore of value in improving our ability to make further inferences about population history and sources of genetic variation. Making phylogenetic predictions on the scale needed for whole-genome analysis is, however, extremely computationally demanding.

**Results:**

In order to facilitate phylogeny-based predictions on a genomic scale, we develop a library of maximum parsimony phylogenies within local regions spanning all autosomal human chromosomes based on Haplotype Map variation data. We demonstrate the utility of this library for population genetic inferences by examining a tree statistic we call 'imperfection,' which measures the reuse of variant sites within a phylogeny. This statistic is significantly predictive of recombination rate, shows additional regional and population-specific conservation, and allows us to identify outlier genes likely to have experienced unusual amounts of variation in recent human history.

**Conclusion:**

Recent theoretical advances in algorithms for phylogenetic tree reconstruction have made it possible to perform large-scale inferences of local maximum parsimony phylogenies from single nucleotide polymorphism (SNP) data. As results from the imperfection statistic demonstrate, phylogeny predictions encode substantial information useful for detecting genomic features and population history. This data set should serve as a platform for many kinds of inferences one may wish to make about human population history and genetic variation.

## Background

Since the first draft sequences of the human genome were completed, much of the sequencing field has turned to the problem of identifying common genomic variations and their distributions among human populations [1-3]. These variations exist predominantly in the form of single nucleotide polymorphisms (SNPs), single DNA bases that take on two common alleles in the population. While most of these variants are believed to be functionally neutral, they nonetheless encode a great deal of information about the history and structure of the population from which they are sampled, as well as the molecular evolution of the local genetic region in which each occurs. Many computational methods have been developed to infer properties of the molecular evolution or population genetics of a species from these SNP data. Examples include methods for identifying sites of frequent recombination (e.g, [4]) or gene conversion (e.g., [5]), identifying conserved haplotype sequences (e.g., [6]), finding genomic regions that have undergone selective sweeps (e.g., [7]), and detecting population substructure (e.g., [8,9]) and admixture (e.g., [10,11]).

All of these inference methods work by a common principle of superimposing a mathematical model of the evolutionary event or process to be detected on a model of neutral evolution in the absence of that process. For example, an inference of population substructure may compare whether observed SNP allele frequencies in the current generation are more consistent with what we expect to find in a single population under Hardy-Weinberg equilibrium or what we expect to see in two genetically isolated populations evolving independently over many generations. Any such inference could in principle be made more easily and accurately if we could observe not just the current generation, but also prior generations at various points in this evolutionary process. Information on these past genetic sequences, commonly encoded in phylogenetic trees or networks, is not generally directly observable but it too can be computationally inferred.

Our goal in this paper is to facilitate a general strategy for performing a range of statistical inferences from genetic variation data: using phylogenetic inferences from the variation data as a common starting point and treating these inferred phylogenies as the input to inferences about specific features of molecular evolution in a population. Similar ideas have previously been applied on smaller scales. Such phylogeny-based inferences have been developed for specific inference problems, such as the detection of likely recombination breakpoints [12]. In addition, genome-scale analyses of phylogenies have been conducted in bacteria. For example, Filliol et al. [13] examined phylogeny inference on a genomic scale for the purpose of categorizing genetic variations from geographically diverse isolates of *Mycobacterium tuberculosis*. Nonetheless, there remain substantial obstacles to the more general use of phylogeny-based inference for whole-genome analysis in eukaryotes. Phylogeny inferences may proceed from a more limited model of molecular evolution than do the downstream inference algorithms and may therefore fail to represent key evolutionary events. Even if the model is correct, the inferred phylogenies may be incorrect. While there is no information in the modern sequences that is not also found in the phylogenies, regardless of their accuracy, incorrect phylogenies may end up confounding our analyses.

Perhaps most limiting is that intra-species phylogeny inference is a computationally demanding task that would be intractable on genomic scales by any widely-used inference method. The simplest variant of the problem is maximum parsimony (MP) [14], which seeks the smallest tree capable of explaining a given data set, a method that tends to be most suitable for short time scales in which mutations are likely to have been infrequent. MP phylogeny inference has been shown to be NP-hard [15] and thus computationally intractable except on small problem instances. There have been some prior methods to solve MP phylogenies optimally, as well as to solve more difficult inferences, such as the inference of phylogenetic networks incorporating both mutation and recombination [16-18]. In practice, MP phylogeny inference is generally performed with fast heuristic methods that do not guarantee optimal solutions (see, for example, Felsenstein [19]). Even these methods are not sufficiently tractable to process the millions of trees one would need for inference on genomic scales. Maximum likelihood [20] and Bayesian [21] methods for phylogeny inference tend to allow for more realistic and detailed mutational models and to more accurately account for uncertainty in inferences than do MP methods, but at the cost of generally even greater computational time.

In the present work, we seek to enable widespread use of phylogeny-based inference for genome-wide analysis by creating a library of local human phylogenies across the human genome drawn from the HapMap variation data [2]. We create this library by applying a recently developed method for maximum parsimony phylogeny inference [22] that made it possible for the first time to construct the millions of local phylogenies needed to enable whole-genome phylogeny-based inferences. We illustrate the use of this library for phylogeny-based inferences with sample applications based on a tree statistic that we call "phylogenetic imperfection." We demonstrate that imperfection shows significant regional and cross-population conservation and show that it is significantly predictive of fine-scale recombination rate.

## Methods

### Phylogeny Inferences

We infer maximum parsimony phylogenies using a method developed in Sridhar et al. [22]. The method finds maximum parsimony mutational phylogenies from matrices of binary SNP variation data. The algorithm first uses a series of preprocessing steps to eliminate redundancy from an observed data set, decompose the problem into smaller sub-problems where possible, and limit the space of possible solutions to each problem. These simplified phylogeny sub-problems are then converted into an equivalent representation based on the graph-theoretic concept of multi-commodity flows. These flow problems then translate directly into a mathematical format called an integer linear program (ILP), a form of constraint satisfaction problem for which highly optimized solver programs are available. We then use the CPLEX 10.0 ILP solver to find an optimal solution to the ILP, which we can then convert into a minimum-size solution to the original phylogeny problem. We refer the reader to Sridhar et al. [22] for details on the theory and implementation of these methods.

### Data Sets

This study primarily uses data from the International Haplotype Map (HapMap Phase II) [2] for the purpose of conducting a fine-scale genome-wide scan of human genetic variations. We use computationally phased HapMap data for this analysis. Although we have developed algorithms that will infer maximum parsimony phylogenies directly from unphased data [23,24], these algorithms are not efficient enough for use on a whole-genome scale. We restricted ourselves to the HapMap CEU population of Utah residents of European ancestry and the YRI population of residents of Yoruba in Ibadan, Nigeria because these subpopulations were genotyped for parent-child trios and can thus be expected to have minimal phasing error. The other two HapMap data sets (Han Chinese in Beijing, China and Japanese in Tokyo, Japan) were genotyped only for unrelated individuals and were omitted here due to the higher likelihood of phasing errors. All HapMap data sets were downloaded in phased form from the HapMap web site, where the PHASE program [25] had been used to identify most likely phases from the trio data. This HapMap build was based on the NCBI human genome assembly build 35 [26]. SNP location assignments and genomic coordinates are therefore based on NCBI build 35. The resulting data contained 120 haplotypes from 60 unrelated individuals for each of the two populations typed at approximately 3.7 million SNPs. Phylogeny inferences were run for window sizes of five, six, seven, eight, and nine consecutive SNPs at each overlapping window of the given size across the 22 autosomal human chromosomes in each of the CEU and YRI populations. The resulting library contains a total of nearly 16 million phylogenies, each covering 5–9 consecutive SNPs on 60 chromosomes. Statistical analyses described below were performed using the 5-SNP libraries. A subset of these analyses, described below, were performed after screening the full library to remove windows spanning predicted recombination hotspots, as assessed by the LDhot method of McVean [4].

Several additional datasets were used to study correlation of imperfection with other sequence features. We retrieved the set of nonsynonymous coding SNPs (ncSNPs) mapped to the build 35 genome using the Ensemble BioMart tool [27,28], selecting all ncSNPs with validated assays. Fine-scale recombination rates and recombination hotspots were retrieved from the HapMap web site [2]. Locations of all short tandem repeats in the human genome were retrieved from the UCSC Genome web site [29]. The set of all human repeats was based on RepeatMasker [30] inferences and was also retrieved from the UCSC Genome resources Table View tool. The locations of high-scoring hits were also manually examined using the UCSC Genome Browser [31] and the dbSNP resource at the NCBI web site [1] to identify the genes and repetitive regions containing the particular SNPs of interest and, for coding SNPs, to identify their corresponding amino acid changes.

### Statistical Analysis

#### Phylogenetic Imperfection

**Figure 1:**
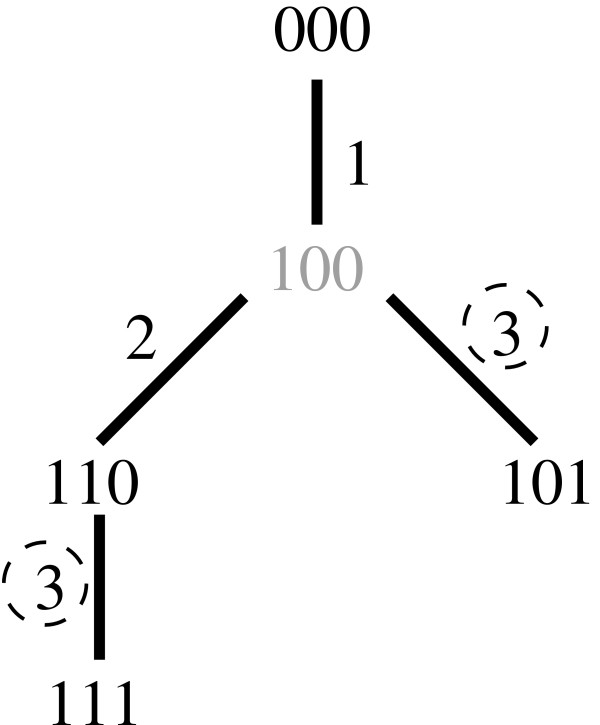
**Illustration of phylogenetic imperfection**. A hypothetical maximum parsimony (MP) tree created from the input sequences 000, 110, 101, and 111, which can be considered binary representations of the alleles at three SNP loci in four individuals. Each edge is labeled with the SNP locus that mutates along that edge. The tree has imperfection 1 because the number of mutations required to explain the tree (4) is one more than the number of variant sites (3), manifesting as two variations in site 3 (dashed circles). Note that imperfection is distinct from the number of unobserved (Steiner) nodes that must be inferred to fit the tree; one such node (100, in grey) is required for this tree. Note also that the MP tree need not be unique for a given data set, but the imperfection score will be the same for all MP trees.

We illustrate the concept of phylogeny-assisted genomic analysis using a simple tree statistic that we call phylogenetic imperfection. The imperfection of a phylogeny is defined as the difference between the number of point mutations found in the tree and the number of variant sites in the data set. In a purely mutational phylogeny, imperfection corresponds to the number of recurrent mutations needed to explain the data set. Figure 1 illustrates the concept of phylogenetic imperfection on a small hypothetical data set. Imperfection can thus be expected to correlate with mutation rate of the SNPs in the tree. It would also be expected to correlate with the presence of recombination or gene conversion, either of which would be expected to be mis-identified as multiple point mutations in a purely mutational phylogeny. In the analysis below, we therefore examine properties of this statistic on a genomic scale, its correlation with recombination rate, and its possible use as a means of identifying sites of high historical variation in the genome.

Note that computing phylogenetic imperfection is NP-hard. While we do not provide a formal proof of that statement, it follows from the fact that knowing the imperfection of a dataset allows one to trivially compute the number of mutations found in the maximum parsimony tree. One could therefore use an efficient algorithm for computing imperfection to create an efficient algorithm for MP phylogeny inference. For example, one might repeatedly identify one possible node and edge on the periphery of an optimal tree whose elimination reduces the optimal parsimony score, then recurse on the remainder of the data to construct the rest of the tree.

#### Mutual information

In order to test for regional variations in phylogenetic imperfection, we calculated mutual information between windows at varying genomic distances. We enumerated all pairs of 5-SNP windows across each chromosome, excluding those with overlapping SNPs. Each pair of windows was placed in a bucket according to the distance separating the central SNPs of the two windows. Bucket widths of 1 kb and 100 kbs were used in separate tests. We then treated the entries in these buckets as samples from two random variables, one variable corresponding to the imperfection of the upstream element of each pair and the other to the imperfection of the downstream element of each pair. We then calculated the mutual information of these two random variables as a measure of how informative an imperfection score at any one point on the genome is about those at varying distances along the genome.

Given a sequence of points *x*_1_,..., *x*_*m *_drawn from a countable set of values {*c*_1_,..., *c*_*n*_}, where *f*_*i *_represents the fraction of points with value *c*_*i*_, the entropy of the sequence is defined as

$$H(x)=-\sum_{i=1}^{n}f_{i}\log(f_{i})$$

The joint entropy of a set of paired data points (*x*_1_, *y*_1_), (*x*_2_, *y*_2_),...,(*x*_*m*_, *y*_*m*_), where *f*_*ij *_is the fraction of points with the value (*c*_*i*_, *c*_*j*_), is calculated by the formula

$$H(x,y)=-\sum_{i=1}^{n}\sum_{j=1}^{n}f_{ij}\log(f_{ik})$$

The mutual information of the set is then defined as *H*(*x*) + *H*(*y*) - *H*(*x*, *y*).

In order to establish statistical significance of mutual information scores, we used the fact that a mutual information score can be regarded as a log likelihood ratio statistic, which itself is approximately chi-square distributed for sufficiently large sample size. Many of the data points in a given bucket will be dependent on one another, even under the null hypothesis that different windows are independent of one another, because the same window may contribute to multiple pairs within a given distance. We therefore adopted a conservative estimate of significance by taking only a subset of pairs containing at most one pair for any given window. With this approximation, the significance of any given data point can be estimated by treating it as a chi-square statistic with value (2 ln 2)*Im*, where *I *is the calculated mutual information and *m *is the number of independent data points supporting it. We calculated this value for each window and used the minimum value of the statistic as an approximate upper bound on the p-value of the comparisons. The number of degrees of freedom is equal to the maximum observed imperfection score, 23 for these data.

#### Imperfection versus recombinations

In order to compare imperfection and fine-scale recombination rates, we first identified for each window in our data set the location of the central SNP in the window. We then retrieved the fine-scale recombination rate at each such SNP from the HapMap-supplied data. The result was two paired lists of data points. We calculated Pearson correlation coefficients for the two lists for each chromosome individually and for all chromosomes collectively. Statistical significance of the correlation coefficients was assessed by permutation test, randomly permuting one data set with respect to the other for 1,000 trials for each test reported. A curve was fit to the data points by proposing that imperfection *i *is related to recombination rate *r *by a function of the form *i *= *a*(1 - *e*^-*br*^) and using Newton-Raphson iteration to find the least-squares best fit parameters *a *and *b*. We calculated the correlation coefficients with the original data-set as well as with windows spanning recombination hotspots removed.

#### Imperfection Outliers

We selected those windows of highest imperfection by summing imperfection computed in the CEU and YRI populations for common windows and selecting those with the highest sum. This analysis was performed using data from which recombination hotspots had been excluded. The analysis was run once for the set of all windows in the genome excluding hotspots and once for those windows centered on non-synonymous coding SNPs (ncSNPs). ncSNPs mapped to the genome were selected from Biomart [27,28] using the February 2006 dbSNP build. We selected all those with combined imperfection at least 24 for the set of all windows and for combined imperfection at least 12 for the set of ncSNP-centered windows. The gene list generated from ncSNP outliers was run through the GOSTAT server [32] to identify overrepresented gene classes. We report only the two most significant such classes as most of the others appear to represent subcategories of those two best hits.

#### Comparison to Maximum Likelihood Phylogenies

One might question whether statistics drawn from our phylogeny library could be biased by systematic errors in phylogeny inference. For example, MP inference can never produce trees larger than the true phylogenies and may therefore systematically underestimate phylogeny size. While there is no known ground truth by which we might definitively test for such biases, we can compare a subset of trees to those from a more statistically sound maximum likelihood (ML) method. Because of the high run-time of ML methods, we can compare only a small subset of the windows. We examined the first 200 5-SNP windows from chromosome 1. We constructed trees for these windows using the Phylip [33] ML inference code, with uniform mutation rate and constant rate variation among sites, using the speedier (S) processing option. We cannot directly compare geometries between the ML and MP trees because the ML approach necessarily treats individuals with the same sequence as distinct tree nodes, creating large subtrees with essentially arbitrary connectivity. We therefore post-process the ML trees by collapsing all nodes of common sequence (both observed haplotypes and inferred Steiner nodes) and relinking the resulting non-redundant node set into a minimum-cost spanning tree. We compared the average phylogeny sizes between these processed ML inferences and our MP data, quantified by the fractional difference between mean MP and mean ML tree size over the 200 data sets. We further quantified differences by two metrics on the geometries of the MP and processed ML trees. We first applied the Robinson-Foulds distance [34], a widely used metric for comparison of phylogenetic trees that measures the number of bipartitions of the population that are defined by edges in one tree but not the other. Second, we examined the root mean square (RMS) difference, summed over all pairs of observed haplotypes, between the separation of the pair in one tree and their separation in the other.

### Computer Resources

The inferences were performed on a Pentium workstation computer running Linux. Code was written in C++ and uses the CPLEX 10 ILP solver for linear programming solution. Our inference algorithms and the phylogeny library are accessible through a web server at . The server provides access to the preprocessed human phylogeny library and a front-end to a server to which users can supply their own data to be solved by our methods. In addition, users can directly download a full set of phylogenies for each chromosome in DOT format, a language for graph description developed for the Graphviz graph rendering package. The present analyses were based on inferences of a single phylogenetic tree per window of SNPs examined, but the server can also infer the network produced by the union of all maximum parsimony phylogenies for any given window. Source code will be provided upon request, although users must supply their own ILP solver to run it.

All other data processing and statistical computations were performed with code written in the Perl language. Graphics for the paper were prepared with SigmaPlot version 10 and Gnuplot version 3.7.

## Results and discussion

### Genome-wide Imperfection Scan

**Figure 2:**
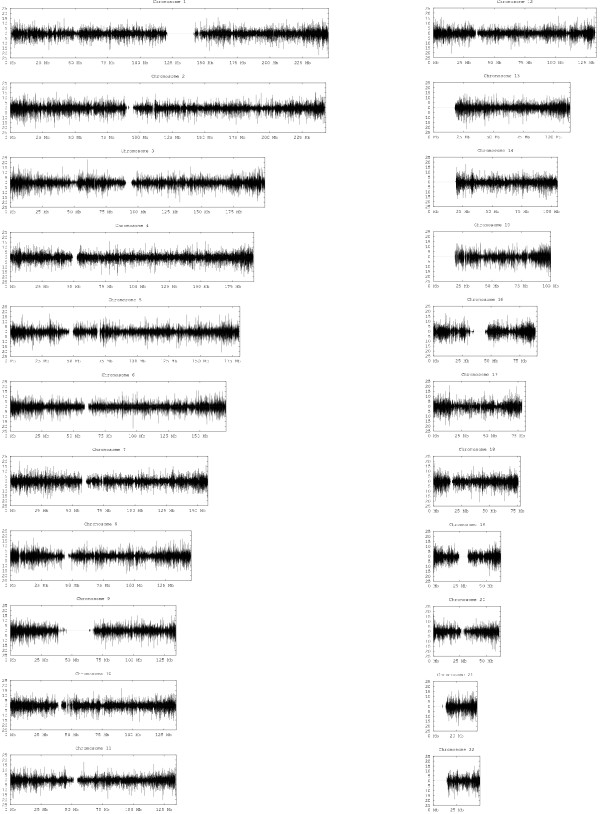
**Genome-wide scans of phylogenetic imperfection**. Each plot shows imperfection for overlapping 5 SNP windows across a single autosomal chromosome. Plots are based on haplotypes determined from trios from the CEU and YRI populations from the HapMap. CEU imperfections are plotted above the x-axis and YRI imperfections below the x-axis.

The principle result of this study is the library of phylogenies, provided for download at . Table 1 provides run-time information for constructing the library. The high variability in run times arises from the fact that run time can vary considerably from tree to tree for a fixed window size and total computational time therefore tends to be dominated from a small fraction of the inferred windows. We cannot directly visualize several million distinct phylogenies and we therefore instead illustrate the overall library by plotting imperfection scores of these phylogenies across the genome for the 5-SNP window library, with windows spanning predicted recombination hotspots removed from the plot. The imperfection score measures the minimal number of recurrent mutations needed to explain mutation among all observed sequences from a common ancestor and therefore provides a simple way of distinguishing regions that can be explained by simple (perfect or near-perfect) phylogenies from those with more complicated histories. Figure 2 presents plots for all human autosomal chromosomes, mapping each window score onto the position of the central SNP in that window. The results show substantial variability in imperfection on multiple length scales. Peaks of high imperfection appear to occur both in isolation and as part of regional clusters. In addition, imperfection appears in general to be higher towards the telomeres and lower towards the centromeres across chromosomes. Imperfection scores around telomeres and centromeres are generally sparse because few SNPs were typed in those regions. Although the amount of data makes it impossible to fully appreciate even the imperfection scores visually, the full set of scores is also available for download at .

**Table 1:** Run times in hours for inferring the complete phylogeny library, separated by window size and population.

Population	5-SNP	6-SNP	7-SNP	8-SNP	9-SNP
CEU	6 h.	16 h.	11 h.	15 h.	128 h.
YRI	13 h.	8 h.	15 h.	37 h.	53 h.

In addition to providing a coarse visualization, the imperfection scores give us a statistic for assessing population conservation of the phylogenies. Over all chromosomes, the CEU data shows a mean imperfection of 0.30 and the YRI a mean imperfection of 0.55. These results may reflect the higher genetic diversity of African versus European populations. The two populations do, however, show a strong overlap in regions of high or low imperfection on the genome. For SNPs variant in both populations, the imperfections have a correlation coefficient of 0.49 between the populations when examining all windows or 0.36 when recombination hotspots are excluded. This correlation may reflect the in fluence of common histories prior to divergence of the two lineages, some inherent propensity of particular sites in the genome towards larger or smaller phylogenies, some combination of the two, or some systematic SNP-specific bias.

### Mutual Information

**Figure 3:**
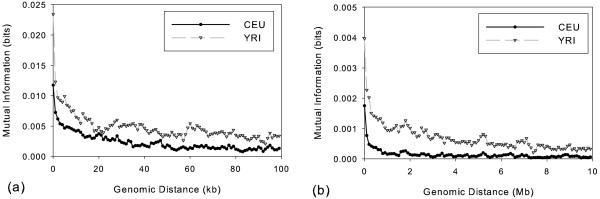
**Dependence of phylogenetic imperfection between windows grouped by distance between the central SNPs of the corresponding windows**. Graphs show mutual information between imperfection levels of non-overlapping windows whose central SNPs fall within a given distance range. (a) Plot of fine-scale correlation, showing mutual information for 1 kb bins up to 100 kb. (b) Plot of coarse-scale correlation, showing mutual information for 100 kb bins up to 10 Mb.

To further test the hypothesis that there is a conserved regional substructure to these patterns of variation, we examined the mutual information between imperfection scores at pairs of non-overlapping windows within each population. Mutual information assesses the degree to which the variability between the two sets of sites treated individually exceeds their variability when considered collectively. High mutual information indicates that two sites are highly predictive of each other, while low mutual information suggests that they are nearly independent. Figure 3(a) shows fine-scale dependence, plotting mutual information for distances 0–100 kb in 1 kb buckets. While both populations yield qualitatively similar results, the YRI population shows in general a somewhat greater mutual information between windows than does the CEU population at all length scales. The plot shows for both populations a sharp spike for the closest windows (0–1 kb apart) followed by a rapid drop and then a slow decline across the remainder of the length range. Figure 3(b) plots dependence at a coarser scale of 0–10 Mb measured in 100 kb buckets. The picture at the coarser scale is qualitatively similar to that at the finer scale, showing a rapid drop for both populations near the origin, followed by a more gradual decline for the remainder of the range covered. The results support the conclusion that imperfection is at least partly determined by an inherent propensity towards high or low imperfection in local regions of the genome. The decaying mutual information scores also help us interpret the observation of conservation across populations. Because mutual information scores are computed in each population separately and using non-overlapping windows, the conservation between nearby windows cannot be explained by the common history of a population prior to divergence into sub-populations or by SNP-specific biases (e.g., a bias due to differential spacing of SNPs on the genome). We can reasonably assume that regional genomic propensities detected by the mutual information measure at least partly explain the conservation of phylogeny sizes between populations. Using the chi-square approximation of mutual information, we determined that the minimum chi-square approximation across all reported mutual information scores was 3270 for 1 kb windows (from the 91 kb bucket) and 152 for 100 kb windows (from the 74 kb bucket). Both values are highly significant (p-value < 10^-6^). We believe this measure provides a very conservative estimate of significance, but it nonetheless establishes that the mutual information between imperfection scores cannot be attributed to chance even out to megabase distances along the genome.

### Imperfection as a Statistic of Fine-scale Recombination

**Figure 4:**
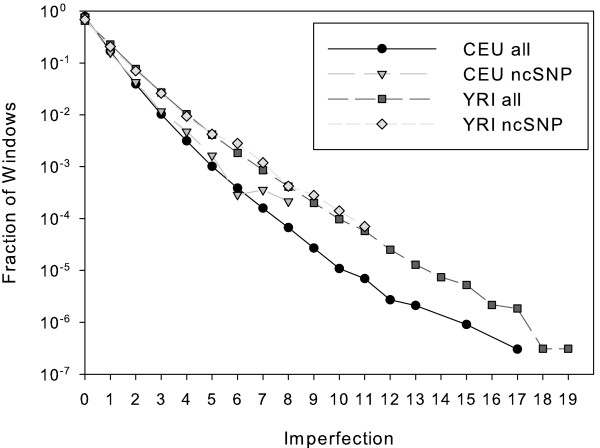
**Coincidence of imperfection and fine-scale recombination rate for chromosome 21**. Imperfection scores are shown as solid grey bars mapped to the position of the central SNP of the corresponding window. Fine-scale recombination rates, supplied by the HapMap web site [2] are marked by dashed black lines. CEU data appear above the x-axis and YRI data below the x-axis.

We next sought to demonstrate how a library of inferred phylogenies would be useful in making genome-scale predictions of genomic features that indirectly depend on local evolutionary histories. We chose the example of fine-scale recombination rate, continuing with the imperfection statistic as a hypothetical predictor of that rate. Recombination rate might be expected to correlate with phylogeny size because recombination events will be misinterpreted as multiple recurrent mutation events and the imperfection statistic should therefore tend to be large where recombination has been frequent. While we do not have access to the ground truth for recombination rate, we can test our ability to predict an accepted inference of the recombination rate that was performed for the HapMap by the method of MacVean et al. [4,35]. Figure 4(a) illustrates the correlation between local imperfection and the previously inferred fine-scale recombination rates for chromosome 21. We chose chromosome 21 for visualization purposes as it is small enough that fine-scale features can still be discerned in a whole-chromosome plot. The image reveals that spikes in local recombination rate do generally correspond with spikes in local phylogenetic imperfection. Conversely, regions of sustained low recombination rate, such as that observed around 28 Mb do appear to correspond to generally low imperfection. Nonetheless, many peaks in phylogenetic imperfection coincide with low inferred recombination rates. Figure 4(b) just examines the windows that fall outside recombination hotspots, further showing that high imperfection can occur at regions of low recombination rates. This observation suggests that the phylogenetic imperfection measure detects both recombination and other sources of large phylogenies, most likely recurrent mutation.

Tables 2 and 3 show the Pearson correlation coefficients between fine-scale recombination rate and phylogenetic imperfection when all windows are included and when windows overlapping recombination hotspots are excluded. Permutation tests verify that the correlations are significant (p < 0.001) for all chromosomes and for both populations. Correlations are nearly identical for the two populations when all SNPs are examined and are slightly higher for YRI when recombination hotspots are excluded. There is a significant correlation regardless of whether or not hotspots are included, but this correlation drops substantially when hotspots are excluded. These observations suggest that phylogeny size is discriminative of recombination rate even within low rates, but that a large fraction of the overall correlation does come from a strong correspondence with recombination hotspots.

**Table 2:** Chromosome-by-chromosome Pearson correlation coefficients of local phylogenetic imperfection and fine-scale recombination rate outside of recombination hotspots.

Chromosome	*r*_*CEU*_^*a *^(CEU)	*r*_*YRI*_^*b *^(YRI)
1	0.23	0.30
2	0.24	0.29
3	0.26	0.30
4	0.24	0.28
5	0.25	0.30
6	0.25	0.29
7	0.26	0.30
8	0.27	0.31
9	0.24	0.30
10	0.24	0.27
11	0.26	0.30
12	0.23	0.28
13	0.26	0.31
14	0.27	0.31
15	0.28	0.32
16	0.28	0.31
17	0.32	0.34
18	0.27	0.34
19	0.32	0.33
20	0.28	0.28
21	0.24	0.27
22	0.29	0.34

(a) Correlation coefficient in the CEU population.

(b) Correlation coefficient in the YRI population.

**Table 3:** Chromosome-by-chromosome Pearson correlation coefficients of local phylogenetic imperfection and fine-scale recombination rate including recombination hotspots.

Chromosome	*r*_*CEU*_^*a *^(CEU)	*r*_*YRI*_^*b *^(YRI)
1	0.37	0.39
2	0.39	0.41
3	0.41	0.41
4	0.39	0.40
5	0.42	0.42
6	0.42	0.41
7	0.41	0.39
8	0.41	0.41
9	0.37	0.38
10	0.38	0.39
11	0.39	0.39
12	0.40	0.38
13	0.42	0.43
14	0.44	0.44
15	0.43	0.41
16	0.38	0.37
17	0.42	0.41
18	0.42	0.45
19	0.38	0.39
20	0.37	0.36
21	0.43	0.41
22	0.39	0.40

(a) Correlation coefficient in the CEU population.

(b) Correlation coefficient in the YRI population.

This analysis also allows us to reconsider the issue of conservation between the populations by examining how well mutual correlation with recombination rate explains correlation of phylogeny sizes between populations. The overall Pearson correlation between recombination and imperfection across all chromosomes is 0.40 for both populations, compared to a correlation of 0.49 between the imperfections of the two populations. These comparisons were likewise all determined to be significant with p-value < 0:001 by permutation tests. When hotspots are excluded, the overall correlations between imperfection and recombination rate drop to 0.36 for both populations. The correlation between imperfection scores for the two populations drops to 0.45. Collectively, these observations confirm that the imperfection scores are significantly influenced by other local genomic properties than just recombination rates.

**Figure 5:**
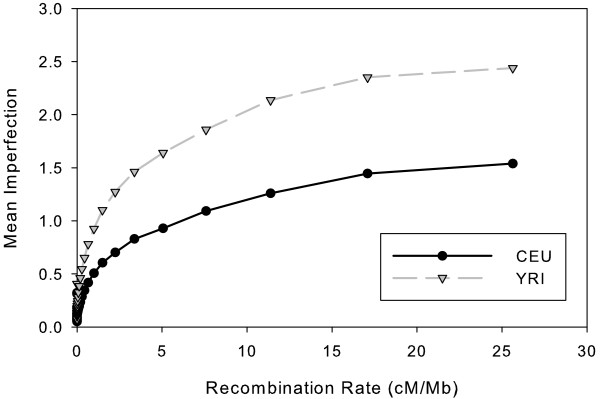
**Dependence of mean imperfection on local recombination rate**. The plot shows mean imperfection scores grouped into binned recombination rates using bins of exponentially increasing size. Each point plots the mean imperfection of a single bin on the y-axis with the lower endpoint of that bin's range on the x-axis.

In order to better understand the relative sensitivity of imperfection to high versus low recombination rates, we plotted a histogram of mean imperfection versus local recombination rate. Given the uneven distribution of data points, we used exponentially increasing bin sizes for recombination rates. After removing bins with fewer than 100 data points, the bin with the smallest rate was (1.5^-26^; 1.5^-25^) and the largest was (1.5^2^; 1.5^3^). Figure 5 presents the results. Both populations show low but non-zero mean imperfection for the smallest recombination rates, rising smoothly with increasing recombination rate before leveling off at the highest rates. The rate of increase and the height of the apparent asymptote varies between the populations, with a more rapid rise and a higher asymptote for YRI (roughly 2.5) compared to CEU (roughly 1.5). We conjectured that the data could be fit by exponentially decaying curves of the form *i *= *a*(1 - *e*^-*br*^). A least-squares fit to this form resulted in the parameters *a *= 0:82 and *b *= 1:02 for the CEU data and *a *= 1:36 and *b *= 1:27 for the YRI data. Note that best-fit curves level off below the asymptote in both cases (*a *is the asymptote), suggesting that a single exponential cannot provide a good fit simultaneously to the low- and high-recombination rate windows. Subtracting out this best fit from the imperfection scores nonetheless reduced the correlation between imperfection and fine-scale recombination from 0.40 to 0.09 for CEU and from 0.40 to 0.11 for YRI while still preserving a correlation of 0.36 between the two populations after the fit was removed. This analysis again confirms that imperfection is strongly predictive of both recombination rate and other sequence regularities distinct from recombination rate.

### Imperfection by SNP Class

**Figure 6:**
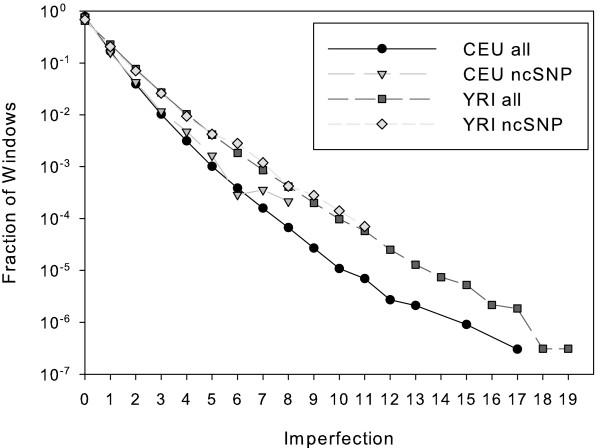
**Histograms of imperfection for windows centered on non-synomous coding SNPs versus all windows**. The plot shows data for all CEU windows (solid line and '+'), all YRI windows (long dash and 'x'), CEU ncSNP windows (medium dash and '*'), and YRI ncSNP windows (short dash and boxes).

We then considered whether the imperfection statistic might be a useful predictor of DNA functional context, such as whether a SNP occurs in coding DNA versus extragenic. We examined this question by comparing the overall distribution of phylogenetic imperfection scores across the genome for various functional classes of windows, with class defined by the sequence context of the central SNP in the window. We first asked whether SNPs likely to be under selection showed any significant bias in imperfection scores. Frequencies of scores were computed for the complete set of windows and for the set of windows centered on validated, non-synonymous coding SNPs, a set chosen because they are likely to be under stronger selection than SNPs in general. Figure 6 shows the results for the CEU and YRI populations respectively. A logarithmic scale is used to allow us to view the full range of frequencies, although this does understate the differences between the four data sets. At a gross level, all four data sets appear quite similar. Each follows an approximately geometric decay in frequency with increasing imperfection. At all imperfections except zero, the fraction of windows for YRI is substantially and consistently larger than that of CEU.

This result is unsurprising since Africans are a more diverse and older population group than Europeans. Low imperfection scores account for a substantial majority of windows for all four sets and the absolute differences between the frequencies at the first few imperfection scores are small. ncSNP windows have a very slightly higher fraction of perfect phylogenies compared to all windows (77.92% versus 77.86% for CEU and 68.02% versus 65.13% for YRI). While there are greater differences between ncSNPs and general windows for larger imperfection scores, these could be explained by the small numbers of examples for the largest imperfection values. Selection against changes in protein code therefore appears to introduce at most a modest bias in window imperfection scores.

**Figure 7:**
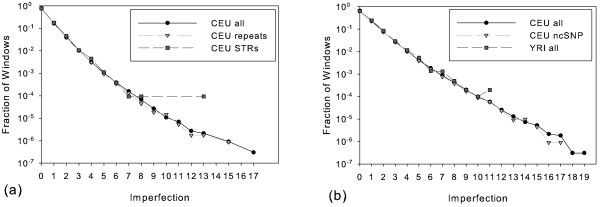
**Histograms of imperfection for repetitive regions versus all windows**. Each plot shows a comparison of windows centered on any repetitive region (solid line and '+'), windows centered on short tandem repeats (long dash and 'x'), and all windows (medium dash and '*'). (a) Data from CEU. (b) Data from YRI.

A bias in imperfection scores might also be expected for SNPs found in repetitive regions of the genome. We might anticipate some excess of imperfection in this set from a greater frequency of genotyping errors or genome misassembly around repetitive elements. We might also anticipate a higher fraction of large imperfection scores due to genuine hypermutable sites, which are known to be associated with some short tandem repeat (STR) regions [36,37]. We therefore compared the set of all windows with those whose central SNP falls in any repetitive region. We also separately examined windows whose central SNPs overlap STR regions. Figures 7(a) and 7(b) show the results for CEU and YRI populations. The graphs show hardly any differences between the data sets for the well-populated imperfection values. Comparing all repeat windows versus all windows, we find that the frequency of perfect windows is nearly identical (77.6% versus 77.9% for CEU and 65.1% in both data-sets for YRI). STR SNPs also do not show pronounced differences from general windows, although they are slightly less likely to be perfect (76.1% versus 77.9% for CEU and 62.3% versus 65.1% for YRI). It therefore appears that repetitive elements do not lead to any dramatic systemic bias in local phylogenetic imperfection.

### Outlier Phylogenies

We finally used the imperfection statistic to demonstrate one final value of a tree statistic in performing whole-genome analysis: identifying outlier data points. There are too many perfect windows to allow for an examination of individual cases of small imperfection, but we can examine those windows of highest imperfection. Based on the preceding analysis, we would expect these outliers to correspond predominantly to windows with high historical recombination. We therefore used the data prescreened to remove recombination hotspots so as to favor outliers produced through processes other than recombination. Table 4 lists the most extreme examples of imperfection observed across the non-hotspot windows. Of the 10 windows, 9 are centered in introns of genes and 1 in an intergenic region. None of the top outliers occurs in the coding region of a gene.

**Table 4:** Windows exhibiting the highest imperfection in the genome.

central SNP^*a*^	chr.^*b*^	pos.^*c*^	*i*_*CEU*_^*d*^	*i*_*YRI*_^*e*^	sequence context
rs2486545	1	240569616	15	17	intergenic region
rs10174559	2	241396766	11	16	intron of KIF1A kinesin family member 1A
rs11683248	2	29360497	11	14	intron of ALK anaplastic lymphoma kinase (Ki-1)
rs7405052	16	84225446	7	17	intron of KIAA0182
rs12918736	16	84226491	7	17	intron of KIAA0182
rs10926263	1	236937389	8	16	intron of FMN2 formin 2
rs6037439	20	296188	17	7	intergenic region
rs7173687	15	24667811	9	15	intron of GABRA5 gamma-aminobutyric acid (GABA) A receptor, alpha 5
rs2493310	1	3317579	9	15	intron of PRDM16 PR domain containing 16
rs8045380	16	84226877	10	14	intron of KIAA0182

This table identifies those windows with combined imperfection scores above 23.

(a) refSNP ID of the central SNP of the window

(b) chromosome on which the window is found

(c) genomic map position of the central SNP

(d) imperfection in the CEU population

(e) imperfection in the YRI population

The results from the previous section suggest the absence of coding SNPs is more likely due to their scarcity in the full SNP set rather than any bias against them and we therefore chose to examine ncSNP outliers separately. Table 5 presents the twelve ncSNP windows yielding the highest imperfection across the genome. The top-scoring hit is to a gene prediction (LOC650137, a predicted olfactory receptor) that may in fact not be a functional gene. The others are each found in known proteins, but for most we know of no reason why they would be particularly disposed to high imperfection. We applied the GOSTAT server [32] to detect overrepresented gene classes among the twelve hits. The two most strongly overrepresented GO classes were GO:0045786 (negative regulation of cell cycle, p-value 0.0119) and GO:0016020 (membrane, p-value 0.0154). Three of the twelve proteins (MTUS1, HEPACAM, and PPP1R15A) are classified as negative cell cycle regulators. Ten of the the twelve (all but PPP1R15A and PRTN3) are membrane-bound. Although three of the twelve are neural-specific – NFASC and NGEF are involved in axonal guidance and HTR3E is a serotonin receptor – this does not appear to be a statistically significant observation. In addition, several of the genes have associations with human disease that might explain unusually strong selection. Four of the twelve occur in genes with known relationships to cancer. MTUS1 encodes multiple transcripts that act as inhibitors of proliferation in many tissue types and appears to be a general tumor suppressor [38]. HEPACAM is frequently downregulated in human hepatocellular carcinoma and may therefore also be a tumor suppressor [39]. TMC8 is a locus for the rare autosomal recessive disease epidermodysplasia verruciformis, which produces high susceptibility to human papillomavirus leading to high risk of skin cancers [40]. PPP1R15A is involved in the response to DNA damage and is a prognostic factor for melanoma progression [41]. One other gene, PRTN3, is normally involved in the inflammation response [42] and is an autoantigen in the autoimmune disease Wegener granulomatosis [43].

**Table 5:** Windows centered on non-synonymous coding SNPs exhibiting the greatest imperfection, excluding recombination hotspots.

central SNP^*a*^	chr.^*b*^	pos.^*c*^	*i*_*CEU*_^*d*^	*i*_*YRI*_^*e*^	gene^*f*^	variation^*g*^
rs1810247	15	19915318	5	11	LOC650137 seven transmembrane helix receptor	C85R
rs17690844	8	17656239	4	10	MTUS1 mitochondrial tumor suppressor 1	T453K
rs2368406	10	29824078	6	8	SVIL supervillin	A809P
rs4973588	2	233660480	4	9	NGEF neuronal guanine nucleotide exchange factor	T111M
rs7208422	17	73642170	8	5	TMC8 transmembrane channel-like 8	I306N
rs3751928	17	68792947	7	6	CDC42EP4 CDC42 effector protein (Rho GTPase binding) 4	
rs7627615	3	185301118	4	9	HTR3E 5-hydroxytryptamine (serotonin) receptor 3, family member E	T86A
rs2802808	1	201698085	4	8	NFASC neurofascin homolog (chicken)	
rs10790715	11	124298892	5	7	HEPACAM hepatocyte cell adhesion molecule	V218M
rs557806	19	54069054	5	7	PPP1R15A protein phosphatase 1 regulatory (inhibitor) subunit 15A	P251R
rs1356410	15	40222129	5	7	PLA2G4F phospholipase A2 group IVF	V740M
rs351111	19	795020	6	6	PRTN3 proteinase 3 (serine proteinase, neutrophil, Wegener granulomatosis autoantigen)	I119V

(a) refSNP ID of the central SNP of the window

(b) chromosome on which the window is found

(c) genomic map position of the central SNP

(d) imperfection in the CEU population

(e) imperfection in the YRI population

(f) gene containing the central SNP of the window

(e) amino acid change produced by the SNP. Annexin A13 has two splice isoforms resulting in two possible sites of variation.

### Comparison to Maximum Likelihood Phylogenies

In order to test for systematic biases in phylogenies introduced by inference from an MP method, we compared them to phylogenies inferred by a maximum likelihood (ML) method for a subset of 200 5-SNP windows. We found a mean phylogeny size of 5.67 mutations for the ML trees versus 5.53 for the MP trees. If we regard the ML trees as a close approximation to the ground truth, then we can conclude MP trees underestimate phylogeny size by an average of 2.5% on these data. We can therefore suggest that, while there is some systematic bias toward smaller trees with an MP method, the bias is relatively modest for small windows. The RMS distance between pairs of individuals for corresponding ML and MP trees is 0.112, suggesting that most individuals are in similar relative positions between the two trees. The Robertson-Foulds distance has a mean value of 0.54, indicative of a somewhat larger average variation when comparing trees by edges rather than by individuals. This mean Robertson-Foulds score can be interpreted as an average of just over one inconsistent edge between each pair of trees. Since we cannot guarantee the optimality of the ML trees by the ML criterion, these measures may in fact overstate the difference between the MP trees and ground truth. It is also possible, though, that the ML trees may themselves be biased relative to the ground truth and may therefore understate the bias in the MP trees. Our post-processing step to collapse sub-trees with identical sequence may also bias the geometries to more closely match those of MP trees. Future examination with other tree statistics or other methods of tree inference may also yield more dramatic differences than we observe here.

## Conclusion

We have used recent methodological improvements in fast phylogeny algorithms to construct a genome-wide library of local mutational phylogenies in the human genome. This library provides an unprecedented view of likely sequences of mutational events in local regions of the genome that may give us new insight into mechanisms of mutation and selective pressures on genomic scales and in individual genes of interest. Many forms of genomic analysis rely on indirect inferences of molecular evolution based on modern observed sequences and would likely benefit from accurate knowledge of full evolutionary histories. While we cannot observe these full histories, it is now possible to make reasonable inferences in local regions. We hope that this library will help enable a "phylogeny first" approach to whole-genome analysis tools based on the common hypothesis that good inferences of phylogenies will provide a stronger basis for statistical prediction of a broad class of genomic features than do raw variation data. We have demonstrated this approach with several sample applications of a simple tree statistic, imperfection, that measures the total size of a phylogeny.

The imperfection tree statistic is predictive of fine-scale recombination rate. It may therefore be useful as an alternative method for estimating recombination rates. Moreover, it detects sequence regularities beyond the correlations for which recombination rate can account, which we conjecture is likely to include local mutation rate biases. Phylogenetic imperfection in conjunction with other measures of recombination rate may be a useful way to separate these possibilities. This result may also in part reflect the fact that recombination rate and mutation rate are themselves correlated [44,45]. It is possible that other mechanisms, such as gene conversion, significantly affect observed imperfection scores. Wiehe et al. [46] showed that one can distinguish recombination and gene conversion by local patterns of linkage disequilibrium (LD); a characteristic pattern of high LD between non-consecutive SNPs with mutual low LD to intervening SNPs might similarly allow one to distinguish genuine recurrent mutation from other possible sources of high phylogenetic imperfection.

The imperfection statistic also allows us to test several hypothesis about molecular evolution on genomic scales. One such hypothesis is that there are significant regional biases in mutational propensities across the genome, beyond what can be accounted for by local signals such as recombination hotspots. Consistent with the hypothesis, imperfection shows a pattern of local correlation on multiple scales, from a strong peak for nearby but non-overlapping windows on the kilobase scale to a gradual decline in correlation on even megabase scales. Given that imperfection strongly correlates with recombination rate but shows significant cross-population correlation even after corrections for recombination rate, it is likely that these regional correlations reflect a combination of regional variation in recombination rate and regional variability in mutation rate. We cannot, however, yet determine the precise degree to which these two factors, or others unknown to us, might contribute to the overall regional variability. Because the information calculations excluded windows sharing SNPs, the very strong local peak on the kilobase scale could only derive from regions extremely dense in SNPs. It is therefore plausible that the fine-scale peak corresponds to local correlations in imperfection due to hypermutable regions of the genome. Further study of regional patterns for known sources of phylogenetic imperfection may help to separate these effects and detect any unanticipated contributing factors. By contrast, the imperfection statistic leads us to reject the hypothesis of significant variations in phylogenetic complexity between coding versus non-coding SNPs, or between SNPs in repetitive versus non-repetitive regions of the genome.

While we use imperfection in the present study as an illustration of our proposed "phylogeny first" approach, there are many other tree statistics that may be informative for particular processes. Gene conversion, selective sweeps, and epistasis, among other processes, might all be anticipated to produce characteristic features of tree geometry by which they might be detected from a phylogeny library. Determining which statistics are informative for particular processes and how they compare to other inference methods is a broad problem that we plan to address in future work.

## Authors' contributions

Both authors participated in all stages of the design, execution, and analysis of the study and in the preparation of this manuscript.
